# Financial Hardship, End-of-Life Health Care Use, and Costs in Patients With Cancer

**DOI:** 10.1001/jamanetworkopen.2026.7923

**Published:** 2026-04-20

**Authors:** Veena Shankaran, Li Li, Sara Khor, Kaiyue Yu, C. Natasha Kwendakwema, Catherine Fedorenko, Karma Kreizenbeck, Hiba M. Khan, Shannon Kestner, Winona Wright, Scott Ramsey

**Affiliations:** 1 Hutchinson Institute for Cancer Outcomes Research, Fred Hutch Cancer Center, Seattle, Washington; 2 Division of Hematology/Oncology, University of Washington School of Medicine, Seattle; 3 Public Health Sciences Division, Fred Hutch Cancer Center, Seattle, Washington; 4 Clinical Research Division, Fred Hutch Cancer Center, Seattle, Washington; 5 The Comparative Health Outcomes, Policy, and Economics (CHOICE) Institute, University of Washington, Seattle; 6 Department of Public Health Sciences, College of Medicine, Pennsylvania State University, Hershey

## Abstract

**Importance:**

Patients with cancer are at higher risk of adverse financial events (AFEs) compared with individuals without cancer. However, little is known about how personal finances affect cancer care, particularly at the end of life (EOL).

**Objective:**

To investigate the association between AFEs and health care use and costs at EOL among patients with cancer.

**Design, Setting, and Participants:**

In this cohort study, Western Washington Surveillance, Epidemiology, and End Results cancer registry cases were linked to claims from commercial payers and Medicare and to credit records from TransUnion. Patients with American Joint Committee on Cancer stage I to IV solid tumors who died between January 1, 2013, and December 31, 2019, were identified. Data analysis was performed from January 2023 and June 2025.

**Main Outcomes and Measures:**

Emergency department (ED) and inpatient visits in the last 3 months of life, place of death, and mean adjusted health care costs per patient were compared between patients with vs without new AFEs in the 2 years preceding EOL (charge-offs, third-party collections, tax liens, delinquent mortgage payments, foreclosures, or repossessions). A multivariate logistic regression analysis evaluated the association between AFEs and the outcomes of ED or inpatient visits and inpatient death. Health care costs in the last 3 and 6 months of life were analyzed using 2-part models to account for zero costs and right-skewed positive expenditures; adjusted average treatment effects were estimated.

**Results:**

A total of 10 826 patients (median [IQR] age, 75 [69-83] years; 5877 [54%] male; 932 [8.6%] with AFEs) were included. Patients with AFEs were more likely to have multiple ED or inpatient visits (odds ratio [OR], 1.41; 95% CI, 1.22-1.62; *P* < .001) and die in a hospital (OR, 1.50; 95% CI, 1.30-1.75; *P* < .001). Mean (SD) adjusted total health care costs were higher in patients with AFEs than those without in the last 3 months ($35 115 [$1415] vs $31 031 [$389]) and 6 months ($57 401 [$2181] vs $51 602 [$581]) of life, yielding a mean adjusted incremental costs in the AFE group of $4084 (95% CI, $1287-$7087; *P* = .006) and $5799 (95% CI, $1235-$9996; *P* = .01), respectively.

**Conclusions and Relevance:**

In this cohort study, AFEs were associated with greater ED and inpatient use, higher risk of inpatient death, and higher care costs at EOL. These findings point to the need for future studies investigating whether interventions that mitigate financial hardship could improve the EOL experience and decrease health care costs for patients with cancer.

## Key Points

**Question:**

Do patients with cancer who struggle financially have more hospital-based and costly care at the end of life?

**Findings:**

This cohort study with 10 826 participants found that patients with cancer with new adverse financial events (eg, collections, liens, foreclosures) in the 2 years before death were 41% more likely to have multiple emergency department or hospital visits in the last 3 months of life, were 50% more likely to die in the hospital, and had higher end-of-life health care spending.

**Meaning:**

These findings suggest that helping patients with cancer who are experiencing financial hardship could improve end-of-life health outcomes and costs.

## Teaser

This cohort study uses credit records to investigate the association of financial strain with end-of-life care and health care spending in a large population-based sample of insured patients with cancer.

## Introduction

Financial hardship (financial toxicity) is now a well-documented consequence of cancer diagnosis, resulting from the cumulative effects of high out-of-pocket medical spending, nonmedical spending (eg, transportation and food), and indirect costs associated with work and income loss. Studies have used a variety of strategies to measure financial hardship, including survey-based methods (incorporating instruments such as the COST-PRO measure), cost diaries, medical bill review, and insurance claims data. A more recent study used consumer credit data to study the impact of cancer diagnosis on financial status (and vice versa) at a population level.^1^ Credit reports provide rich and objective financial data that are regularly updated and available for up to 90% of US residents, including socioeconomically disadvantaged and elderly populations.^2,3,4^ Using a novel database linking cancer registry, bankruptcy, and consumer credit data in Washington State, previous studies found that patients with cancer are 2.65 times more likely to file for bankruptcy and 1.71 times more likely to experience a major adverse financial event (AFE), such as a third-party collection, charge-off, lien, delinquent mortgage payment, foreclosure, or repossession, compared with similar matched individuals without cancer.^1,5^

Several previous studies have highlighted the negative impacts of cancer-related financial hardship, including poorer quality of life, lower treatment adherence, and worse survival.^6,7,8,9,10^ However, few studies have reported on the association between financial hardship and the type of care patients receive at the end of life (EOL), a period that is the most medically, logistically, financially, and emotionally challenging time for patients and families. From a health system standpoint, EOL care is also associated with the highest overall health care spending; approximately one-quarter of Medicare spending is incurred by individuals in their last year of life, driven in large part by hospital-based care.^11,12,13^ In oncology, this is exacerbated by a trend toward increased use of high-cost therapeutics at EOL.^14^

Although previous research^30,31^ has shown that patients with cancer from minoritized race and ethnicity groups and those with Medicaid insurance have a higher likelihood of receiving more intense care at the EOL, little is known about the association among financial strain, EOL care, and costs. A 2015 study found that self-reported financial hardship was associated with higher-intensity care in the last week of life, after adjusting for sociodemographic factors, such as race, ethnicity, and insurance type.^15^ This study,^15^ however, was limited by a small cohort (n = 281) and a single self-reported measure (financial hardship). The goal of the current study is to further investigate the association between financial strain using credit records, EOL care, and health care spending in a large population-based sample of insured patients with cancer.

## Methods

### Study Design

We used a retrospective cohort study design to investigate the association among financial hardship as identified in credit records (hereafter referred to as AFEs), EOL health care use, and total health care costs. Our central hypothesis is that patients with cancer who experienced new AFEs in the last 2 years of life were more likely to have multiple emergency department (ED) or hospital visits at EOL, were more likely to die in the hospital, and had higher overall total health care spending at EOL. Institutional review board approval for this study was granted by the Fred Hutch Cancer Center. The study was deemed minimal risk and a waiver of informed consent was granted. A data use agreement was previously established between TransUnion and the Fred Hutch Cancer Center detailing data safety, privacy, storage, and sharing procedures for both entities. This study followed the Strengthening the Reporting of Observational Studies in Epidemiology (STROBE) reporting guideline for cohort studies.

### Data Sources and Linkage

The data source for this study was a unique database that links cancer registry data from the Western Washington Surveillance, Epidemiology, and End Results (SEER) registry with claims from the major health payers in Washington State (Premera, Regence, and Medicare) with quarterly credit records from TransUnion, 1 of 3 large credit agencies in the US. Credit records were provided as quarterly records on all participants, starting in quarter 3 of 2012 through quarter 2 of 2020 such that each patient had credit data available at diagnosis and death. This database and linkage have been previously described.^1^

### Patient Population

The cohort consisted of patients with American Joint Committee on Cancer (AJCC) stage I to IV (excluding stage 0) solid tumors who were 18 years or older with commercial or Medicare (or both) insurance diagnosed between January 1, 2013, and December 31, 2019, who died (of any cause) during this same period. Patients were excluded if they died within 3 months of diagnosis or did not have continuous enrollment in Medicare or a commercial insurance plan in the last 6 months of life. Medicaid and dual Medicaid-Medicare enrollees were not included given high correlation between financial hardship and Medicaid eligibility. Race and ethnicity categories included Alaska Native or American Indian, Asian or Pacific Islander, Black, White, or multiple races or unknown.

### Adverse Financial Events

The main variable in the regression model was the presence of a new AFE in any of the credit reports between 3 and 24 months prior to death. Credit data in the last 3 months of life were not included to avoid measuring the variable and outcome simultaneously. AFEs were defined as evidence of foreclosure trades, repossession trades, tax liens, delinquent mortgage trades, charge-off trades, and medical and nonmedical third-party collections. This definition of AFE is consistent with a prior study showing that patients with cancer have a higher risk of AFEs than matched individuals without cancer.^1^ New AFEs in the 3 to 24 months prior to death was defined as developing an AFE having not previously had one or a numerical increase in the number of any AFEs (eg, from 0 to 1 delinquent mortgage payments or from 3 to 4 collections). AFEs were categorized by levels of severity starting with (1) inability to pay bills, manifesting as collections or charge-offs; (2) tax liens and delinquent mortgage payments that put individuals at risk for creditor action against their property; and (3) foreclosures and repossessions.^1^

### Health Care Utilization Outcomes

The primary outcome was claims for multiple (>1) ED visits or inpatient hospitalizations during the last 3 months of life. A 3-month EOL window was chosen because it has been identified to be most sensitive to escalation patterns that may reflect fragmented, costly, or non–goal-concordant care.^16,17,18,19,20^ The secondary outcome was place of death identified in SEER, dichotomized as death in the hospital setting (ED or inpatient) or outpatient setting (hospice, home, nursing home, or other).

### Health Care Costs

Total health care costs were determined for all patients in the last 3 and 6 months of life by totaling costs for all paid claims during these periods. Patient out-of-pocket spending could not be accurately estimated and thus was not included in the estimation of total health care costs.

### Statistical Analysis

We used descriptive statistics (means, medians, and proportions) to summarize patient characteristics and determine the proportion experiencing new AFEs in the 3 to 24 months prior to death. We used multivariate logistic regression analyses to study the association of new AFEs with our primary outcome of multiple ED and inpatient hospital visits and our secondary outcome of hospital death (2-sided α = .05). Our analyses were adjusted for baseline demographic and clinical characteristics, including age, sex, race, Charlson Comorbidity Index, payer type, marital status, AJCC stage at diagnosis (dichotomized as early stage [stage I or II] and late stage [stage III and IV]), presence of AFEs at baseline (24 months before death), and Area Deprivation Index (ADI), which is a composite measure of neighborhood socioeconomic status across 10 categories, with higher values reflecting more deprived neighborhoods.^21^ We categorized ADI into 3 groups; low ADI (score,1-3), moderate ADI (score, 4-7), and high ADI (score, 8-10). In addition, we adjusted for diagnosis year and the start of the AFE window (24 months prior to death) because both may influence AFE risk via out-of-pocket spending and economic conditions during that period (eAppendix in Supplement 1).

Health care costs in the last 3 and 6 months of life were analyzed using 2-part models to account for zero costs and right-skewed positive expenditures. First, we estimated the probability of incurring any costs using multivariable logistic regression. Second, among individuals with positive costs, we modeled expenditures using a generalized linear model with a γ-distribution and log link. Both parts adjusted for prespecified baseline covariates and the exposure of interest. Adjusted average treatment effects were estimated via marginal standardization, and 95% CIs were obtained using 1000 bootstrap resamples.

We conducted sensitivity analyses investigating the association between AFEs and our outcomes in the population with late-stage cancer (stage III and IV) only and in patients who were known to have cancer-related cause of death. In addition, as a robustness check, we one-to-one matched pairs of patients from the group that experienced new AFEs from 3 to 24 months before death and the group who did not experience new AFEs by using their propensity scores and a caliper equal to one-quarter of the SD of the logit of the propensity score, matching on all variables used in the regression analysis. We then used a logistic regression in the propensity score–matched sample to determine the risk of ED or hospital use and inpatient death. Data analysis was performed from January 2023 and June 2025.

## Results

### Patient Characteristics

The final study cohort consisted of 10 826 patients (median [IQR] age, 75 [69-83] years; 5877 [54%] male and 4949 [46%] female; 67 [<1%] American Indian or Alaskan Native, 357 [3%] Asian or Pacific Islander, 166 [2%] Black, 10 071 [93%] White, and 165 [2%] multiracial or unknown race; 932 [8.6%] with AFEs) who met the eligibility criteria (Table 1). Most patients were 65 years or older (9342 [86%]), Medicare insured (7485 [69%]), and partnered (5839 [54%]). A total of 1902 patients (17%) came from the most socioeconomically deprived neighborhoods (ADI scores, 8-10). Most patients had advanced-stage (III or IV) cancer (4960 [46%]) or unstaged cancers (2128 [20%]), which tend to have similar outcomes as higher-stage cancers. The most common cancers were lung cancer (2482 [23%]) followed by breast (814 [8%]) and colorectal cancer (922 [9%]). A total of 7718 patients (72%) had cancer-specific cause of death.

**Table 1.** Patient Characteristics

Characteristic	No. (%) of patients (N = 10 826)
Sex	
Female	4949 (46)
Male	5877 (54)
Age group, y	
≥65	9342 (86)
<65	1484 (14)
Race	
American Indian or Alaskan Native	67 (<1)
Asian or Pacific Islander	357 (3)
Black	166 (2)
White	10 071 (93)
Multiracial or unknown	165 (2)
Charlson Comorbidity Index	
0	4762 (44)
1	2423 (22)
>1	3687 (34)
Marital status	
Living without a partner	4015 (37)
Living with a partner	5839 (54)
Unknown	972 (9)
Payer type	
Medicare	7485 (69)
Commercial (Regence or Premera)	1248 (12)
Multiple insurance	2093 (19)
ADI score	
1-3	3964 (37)
4-7	4960 (46)
8-10	1902 (17)
AJCC stage	
I	2099 (19)
II	1662 (15)
III	1772 (16)
IV	3165 (29)
Unstaged	2128 (20)
Cancer type	
Bladder	481 (4)
Breast	814 (8)
Colorectal	922 (9)
Gynecologic	565 (5)
Kidney	404 (4)
Liver	251 (2)
Lung	2482 (23)
Melanoma	448 (4)
Oral	342 (3)
Pancreas	727 (7)
Prostate	827 (8)
Thyroid	65 (<1)
Other	2498 (23)

Abbreviations: ADI, Area Deprivation Index; AJCC, American Joint Committee on Cancer.

### Adverse Financial Events

In this cohort, 932 patients (8.6%) had a new AFE from 3 to 24 months prior to death. The most common type of AFE was third-party collections (701 [6.5%]). A total of 1158 patients (10.7%) had evidence of a baseline preexisting AFE 24 months prior to death. Repossessions and foreclosures were uncommon (Table 2).

**Table 2.** AFEs in the Study Population

AFE	No. (%) of patients (N = 10 826)
Evidence of baseline AFE	1158 (11)
New AFE (between 3 and 24 mo before death)	932 (9)
Severe AFEs	823 (8)
Third-party collections	701 (6)
Charge-offs	258 (2)
More severe AFEs	152 (1)
Tax liens	18 (<1)
Delinquent mortgage payments	134 (1)
Most severe AFEs	31 (<1)
Foreclosure	9 (<1)
Repossessions	22 (<1)

Abbreviation: AFE, adverse financial event.

### Health Care Use 

A total of 3476 patients (32%) experienced multiple ED or inpatient hospitalizations in the last 3 months of life, and 2727 patients (25%) died in the hospital. A total of 7718 patients (72%) died of cancer; cause of death was cancer-related in 2072 patients (55%) with stage I to II disease and 4283 patients (88%) with stage III to III disease. Patients with a new AFE between 3 and 24 months prior to death were significantly more likely to experience multiple ED or hospital visits at EOL (odds ratio [OR], 1.41; 95% CI, 1.22-1.62; *P* < .001) and to die in the hospital (OR, 1.50; 95% CI, 1.30-1.75; *P* < .001) (Table 3).

**Table 3.** Multivariable Logistic Regression Results Examining the Association Between AFE and Multiple ED and Hospital Visits and Place of Death

Factor	OR (95% CI)	*P* value
**Factors associated with multiple ED and hospital visits**		
AFEs		
No AFE	1.00 [Reference]	NA
Any AFE	1.41 (1.22-1.62)	<.001
Age group, y		
≥65	1.00 [Reference]	NA
<65 y	1.25 (1.08-1.43)	.002
Sex		
Female	1.00 [Reference]	NA
Male	1.04 (0.95-1.13)	.40
Marital status		
Unpartnered	1.00 [Reference]	NA
Partnered	1.18 (1.07-1.29)	<.001
Marital status unknown	1.13 (0.97-1.32)	.12
Charlson Comorbidity score		
0	1.00 [Reference]	NA
1	1.11 (1.00-1.24)	.053
2	1.22 (1.11-1.34)	<.001
Race		
Alaska Native or American Indian	1.16 (0.72-1.88)	.56
Asian or Pacific Islander	0.95 (0.76-1.19)	.66
Black	1.06 (0.76-1.46)	.75
White	1.00 [Reference]	NA
Multiracial or unknown	0.84 (0.60-1.18)	.33
AJCC stage		
Early stage (stages I-II)	1.00 [Reference]	NA
Advanced stage (stages III-IV)	1.00 (0.91-1.10)	.93
Unstaged	0.83 (0.74-0.94)	.002
Payer type		
Medicare	1.00 [Reference]	NA
Commercial	1.00 (0.86-1.17)	.99
Multiple insurance	1.07 (0.96-1.19)	.20
ADI score		
0-3	1.00 [Reference]	NA
4-7	1.07 0.98-1.18)	.12
8-10	1.10 (0.98-1.24)	.10
Diagnosis year	1.07 (1.03-1.10)	<.001
Start year of new AFE exposure	0.97 (0.95-1.00)	.10
**Factors associated with place of death in the hospital**		
AFEs		
No AFE	1.00 [Reference]	NA
Any AFE	1.51 (1.30-1.75)	<.001
Age group, y		
≥65	1.00 [Reference]	NA
<65	1.17 (1.01-1.36)	.04
Sex		
Female	1.00 [Reference]	NA
Male	1.05 (0.96-1.15)	.27
Marital status		
Unpartnered	1.00 [Reference]	NA
Partnered	1.18 (1.07-1.30)	.001
Marital status unknown	1.05 (0.88-1.23)	.60
Charlson Comorbidity score		
0	1.00 [Reference]	NA
1	1.32 (1.19-1.49)	<.001
2	1.32 (1.19-1.46)	<.001
Race		
Alaska Native or American Indian	1.18 (0.69-2.00)	.55
Asian or Pacific Islander	1.06 (0.83-1.36)	.62
Black	1.24 (0.89-1.74)	.21
White	1.00 [Reference]	NA
Multiracial or unknown	1.09 (0.77-1.53)	.64
AJCC stage		
Early stage (stages I-II)	1.00 [Reference]	NA
Advanced stage (stages III-IV)	0.77 (0.69-0.85)	<.001
Unstaged	0.83 (0.73-0.94)	.003
Payer type		
Medicare	1.00 [Reference]	NA
Commercial	1.17 (0.99-1.38)	.05
Multiple insurance	0.94 (0.84-1.06)	.33
ADI		
0-3	1.00 [Reference]	NA
4-7	0.95 (0.86-1.05)	.31
8-10	1.09 (0.96-1.24)	.17
Diagnosis year	1.00 (0.97-1.04)	.89
Start year of new AFE exposure	1.04 (1.01-1.08)	.009

Abbreviations: ADI, Area Deprivation Index; AFE, adverse financial event; AJCC, American Joint Committee on Cancer; ED, emergency department; NA, not applicable; OR, odds ratio.

### Sensitivity Analyses

In a sensitivity analysis focusing on advanced-stage (stage III and IV) disease only, AFEs were associated with multiple ED or inpatient hospitalizations (OR, 1.29; 95% CI, 1.0-1.59; *P* = .01) and dying in the hospital (OR, 1.52; 95% CI, 1.2-1.89; *P* < .001). In the 7718 patients (72%) known to have a cancer-related death, AFEs were associated with an increased risk of multiple ED or inpatient hospitalizations (OR, 1.30; 95% CI, 1.09-1.54; *P* = .003) and dying in the hospital (OR, 1.55; 95% CI, 1.28-1.87; *P* < .001). The propensity score–matched sample consisted of 1852 patients (926 patients with a new AFE and 926 without a new AFE). In the propensity score–matched sample, a new AFE was also associated with an increased risk of multiple ED or inpatient hospitalizations (OR, 1.38; 95% CI, 1.14-1.66; *P* = .001) and dying in the hospital (OR, 1.39; 95% CI, 1.14-1.70; *P* = .001).

### Health Care Costs

Mean (SE) adjusted costs in the last 3 months of life were $35 115 ($1415) among patients who experienced new AFEs vs $31 031 ($389) in patients who did not, yielding an adjusted average treatment effect of $4084 (95% CI, $1287-$7087; *P* = .006) in the AFE group (Figure). Similarly, mean (SE) adjusted costs in the last 6 months of life were $57 401 ($2181) among patients with a new AFE vs $51 602 ($581) in those without, for an average treatment effect of $5799 (95% CI, $1235-$9996; *P* = .01).

**Figure.**
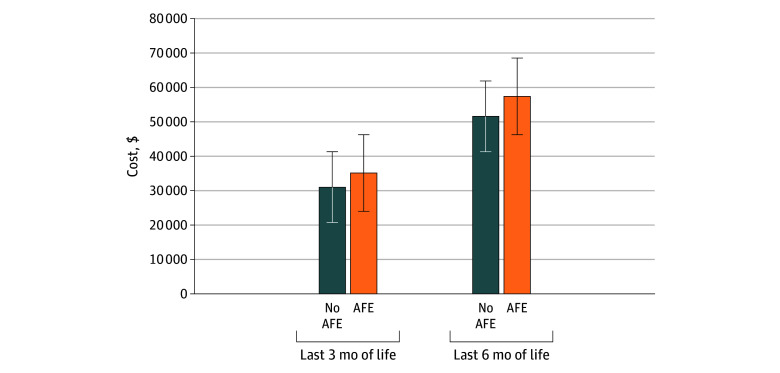
Bar Graph of the Mean Adjusted Per-Patient Costs at End of Life for Patients With and Without an Adverse Financial Event (AFE) Error bars indicate SDs.

To account for multiple comparisons, we controlled the false discovery rate using the Benjamini-Hochberg procedure (false discovery rate = .05). All 4 *P* values satisfy the Benjamini-Hochberg rejection criterion; thus, all null hypotheses are rejected after adjustment.

## Discussion

Among patients with cancer who died, presence of an AFE before the EOL period was associated with higher health care utilization (ED and hospital use) at EOL and greater risk of dying in the hospital. In addition, EOL health care costs (claims paid by the insurer) were higher in patients who had experienced new AFEs leading up to the EOL period. This is one of the first studies, to our knowledge, to use objective population-based financial data to show an association between financial hardship and health care use and costs at EOL in patients with cancer. Whereas prior research on the downstream effects of cancer-related financial hardship have focused on patient outcomes such as quality of life and mortality, this study looks at health system–level outcomes such as health care use and direct medical care costs, giving additional support to the need for interventions to support patients who are struggling with financial hardship. Prior research suggests that lay patient navigation (including logistic and financial navigation) may help to reduce resource use and costs among patients with cancer who receive Medicare.^22,23^ This foundational work on the potential for navigation to influence health care use should motivate future studies that investigate the impact of financial navigation on EOL outcomes. In addition to the obvious burden on patients and families who spend the last months of their life in the hospital, the implications in terms of higher health care costs may provide a larger societal case for addressing health-related social needs and financial hardship in patients with cancer.

Although our study does not elucidate the mechanisms underlying our findings, we hypothesize that patients with AFEs may be more likely to use the ED and hospital at EOL due to factors such as difficulty affording home caregivers, inability to afford copayments for supportive care medications and other treatments, and difficulty coming to clinic visits to discuss symptom management and goals of care. AFEs may also be a larger indicator of poor health literacy, social risk, adverse social determinants of health, and health-related social needs, which are known to be associated adverse clinical outcomes, including higher ED and hospital use.^24,25,26^ Although our analyses adjusted for neighborhood-level socioeconomic status (ADI), we did not have access to information about household income, wealth, assets, health literacy, social networks, and access to housing, shelter, and food, all of which are likely associated with new AFEs.

### Limitations

Several limitations of this study should be acknowledged. Our cohort is not limited to patients with advanced-stage disease, and the cause of death is not limited to cancer. Given differences in the approach to EOL care for patients with cancer vs other diseases, cause of death may explain why patients with later-stage cancer were less likely to die in the hospital compared with patients with earlier-stage disease. When focusing, however, on only the patients with later-stage disease, we similarly see associations between AFE and our outcomes of interest. We also see similar associations when limiting our analyses to those whose cause of death was cancer. We believe that our findings suggest that AFEs among cancer survivors are a risk factor for increased health care use at EOL, regardless of cancer stage or cause of death. In addition, if these AFEs were caused by the cancer treatments patients had received, the implication that these AFEs might prevent patients from accessing future outpatient care, particularly at EOL, is notable. Indeed, our findings may have larger implications across various health conditions. Another limitation of this study is the lack of mechanistic explanation of the observed association. Although we hypothesize that difficulty accessing outpatient and home care might play a role, we are not able to show this using our data. Future studies should explore the reasons behind the association between financial hardship and EOL health care utilization. Next, the study population comes from Western Washington, a predominantly urban and relatively wealthy region of the US; our findings are not necessarily generalizable to the entire US, particularly to more rural areas. Moreover, our findings are not generalizable to uninsured patients or Medicaid enrollees, and we acknowledge that our study may underestimate the burdens faced by uninsured or Medicaid individuals at EOL as noted in several prior studies.^27,28^ Another limitation is that the severe AFEs that we studied are relatively rare and do not represent the complete set of possible financially harmful events that patients may experience, including psychological and behavioral impacts (eg, financial worry and treatment nonadherence). We also recognize that individuals who do not own property are not eligible for certain AFEs, specifically, foreclosures, tax liens, and repossessions. Thus, although our study shows that very specific financial hardships may be associated with EOL ED and hospital use, we cannot conclude that the same is true for all types of financial hardships.

Despite these limitations, to our knowledge, our study is one of the first to show, at a population level, an independent association between financial hardship and EOL health care use and costs using objective and standardized consumer credit data and adjusting for a variety of other factors that might also influence these outcomes. EOL care patterns may therefore represent a culmination of events, experiences, and hardships that start early in the disease trajectory. Although goals-of-care conversations are certainly a cornerstone for improved EOL care, addressing other financial barriers and health-related social needs that might play into the EOL experience may also be essential. Ongoing financial interventional studies, including CREDIT,^29^ will contribute to this goal by exploring the impact of financial navigation on various outcomes including ED and hospital use in patients with advanced cancer. Further understanding of the specific pathway between financial hardship and EOL health care use could help inform more targeted interventions.

## Conclusions

Previous work using consumer credit data to estimate the relative risk of financial hardship in patients with cancer paved the way for the current study, which found an association between financial hardship and EOL care and costs. This additional dimension regarding financial toxicity in oncology further underscores the need for strategies at the clinic, health system, and policy levels to mitigate the financial burden of cancer treatment.

## Notes

Supplement 1. **eAppendix. **Directed Acyclic Graph (DAG) Relating Adverse Financial Events to End-of-Life Healthcare Utilization and Costs

Supplement 2.
Data Sharing Statement
