# Supplementary material for: Financial Hardship, End-of-Life Health Care Use, and Costs in Patients With Cancer
Source: JAMA Netw Open. 2026 Apr 20;9(4):e267923. doi: 10.1001/jamanetworkopen.2026.7923 (PMC13096980; doi:10.1001/jamanetworkopen.2026.7923)
Supplement: Supplement 1. — eAppendix. Directed Acyclic Graph (DAG) Relating Adverse Financial Events to End-of-Life Healthcare Utilization and Costs [file jamanetwopen-e267923-s001.pdf]

Supplemental Online Content

Shankaran V, Li L, Khor S, et al. Financial hardship, end-of-life health care use, and costs in patients with cancer. *JAMA Netw Open*. 2026;9(4):e267923.
doi:10.1001/jamanetworkopen.2026.7923

eAppendix. Directed Acyclic Graph (DAG) Relating Adverse Financial Events to End-of-Life Healthcare Utilization and Costs

This supplemental material has been provided by the authors to give readers additional information about their work.

© 2026 Shankaran V et al. *JAMA Network Open*.

eAppendix. Directed Acyclic Graph (DAG) Relating Adverse Financial Events to End-of-Life Healthcare Utilization and Costs

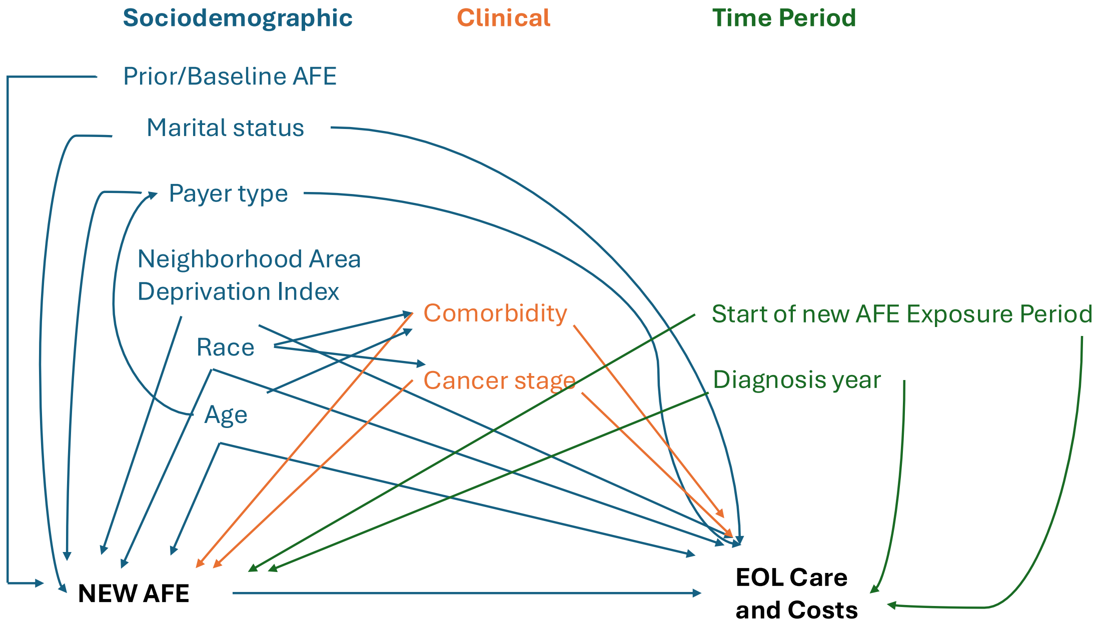

© 2026 Shankaran V et al. *JAMA Network Open*.
