# Supplementary material for: Financial Hardship, End-of-Life Health Care Use, and Costs in Patients With Cancer
Source: JAMA Netw Open. 2026 Apr 20;9(4):e267923. doi: 10.1001/jamanetworkopen.2026.7923 (PMC13096980; doi:10.1001/jamanetworkopen.2026.7923)
Supplement: Supplement 2. — Data Sharing Statement [file jamanetwopen-e267923-s002.pdf]

Data Sharing Statement

Shankaran. Financial Hardship, End-of-Life Health Care Use, and Costs in Patients With Cancer. *JAMA Netw Open*. Published April 20, 2026. doi:10.1001/jamanetworkopen.2026.7923

Data
Data available: No

Additional Information
Explanation for why data not available: Data use agreements with payers / credit agency prohibits data sharing
